# Preferences of Healthcare Professionals on 3D-Printed Tablets: A Pilot Study

**DOI:** 10.3390/pharmaceutics14071521

**Published:** 2022-07-21

**Authors:** Odelia Goh, Wei Jiang Goh, Seng Han Lim, Grace S. Hoo, Raymond Liew, Tat Ming Ng

**Affiliations:** 1 School of Materials Science & Engineering, Nanyang Technological University, Singapore 639798, Singapore; ogoh003@e.ntu.edu.sg; 2 Craft Health Pte, Ltd., 83 Science Park Drive, Singapore Science Park 1, The Curie, #03-01A, Singapore 118258, Singapore; weijiang.goh@crafthealth.me (W.J.G.); senghan.lim@crafthealth.me (S.H.L.); 3 National Centre for Infectious Diseases, Department of Pharmacy, Singapore, 16 Jalan Tan Tock Seng, Singapore 308442, Singapore; grace_sr_hoo@ncid.sg; 4 Division of Pharmacy, Tan Tock Seng Hospital, 11 Jalan Tan Tock Seng, Singapore 308433, Singapore; raymond_liew@ttsh.com.sg

**Keywords:** 3D printing, polypill, medication adherence, healthcare professional perceptions

## Abstract

**Highlights:**

**1)**  **What are the main findings?**
  - More than 70% of the respondents agreed about the benefits of 3D printed tablets.
  - More than 60% of the respondents were willing to prescribe 3D printed tablets.
  - Many of the respondents were concerned about formulation considerations, manufacturing processes, and administrative issues.
**2)**  **What is the implication of the main finding?**
  - Healthcare professionals are receptive to adopting 3D printed tablets.
  - Addressing formulation, manufacturing process and administrative concerns is important for technology adoption.
  - 3D printing companies, healthcare professionals and regulators should closely collaborate to advance the benefits of 3D printed tablets.

**Abstract:**

An inaugural study was performed to understand the perceptions of healthcare professionals toward the potential benefits of 3D printing in Singapore. This study sought to increase awareness of 3D printing applications for viable clinical applications and to elucidate the current gaps in therapy where 3D printing could play a role. A common example would be the use of 3D printing to manufacture polypills, thereby reducing the daily pill burden of patients and possibly improving medication adherence. A qualitative descriptive survey with a single-centered cross-sectional design was performed at Tan Tock Seng Hospital, a tertiary referral hospital with 1700 beds. This study had a total of 55 respondents comprising doctors and pharmacists. Most of the respondents viewed the 3D printing of oral dosage forms favorably and agreed about the potential advantages this technology could offer. More than 60% of the respondents were also willing to prescribe 3D printed tablets to patients. Respondents’ concerns were grouped into three main categories: formulation considerations, manufacturing processes, and administrative issues. Viewed in its entirety, this study provides a valuable starting point for understanding the perceptions of healthcare professionals in adopting 3D printing technology.

## 1. Introduction

Many patients, especially those with chronic diseases, present with complex dosing regimens [1], including polypharmacy (most commonly defined as needing to take five or more medications daily) [2], high frequency of medicine-taking, and daily dosage adjustments. This complexity results in a challenging environment for healthcare professionals, caregivers, and patients to ensure medication adherence, which can lead to poor patient outcomes [3]. Additionally, there is a significant prevalence of multiple chronic conditions (MCC) globally, with approximately one in three adults having two or more chronic conditions [4], which further increases the complexity of medication regimens.

The emerging technology of 3D printing for the manufacture of pharmaceuticals has the potential to simplify medication regimens and to overcome such challenges. With the development of various techniques, such as semi-solid extrusion (SSE), binder jetting (BJ), and fused deposition modeling (FDM) [5], 3D printing technology for pharmaceutical manufacturing has advanced from a theoretical concept to actual clinical applications, complementing conventional manufacturing processes. Unlike traditional mass-manufacturing technologies such as tableting and encapsulation, 3D printing offers the flexibility of “on-demand” printing and the ability to personalize medications according to a patient’s needs. Different geometries, release characteristics, unique dosages, and even a combination of multiple drugs can be obtained and adjusted easily with 3D printing [6]. Table A1 (Appendix A) highlights the benefits and impacts of 3D printing for the healthcare sector, with examples of applications.

However, the adoption of 3D printed tablets in clinical settings relies not only on technological advances but also on the acceptance of 3D printing by key stakeholders, including prescribers and patients. While there are studies on patients’ preferences and acceptance of 3D printed tablets [7,8], there has only been one study regarding the perceptions of healthcare professionals regarding 3D printed medicines in a pediatrics hospital [9]. In addition, many pharmacists may still not be aware of 3D printing for personalized medicines [10]. Hence, this study—one of the first to be conducted in Singapore and the surrounding region—seek to further inform on the potential adoption of 3D printed tablets in Singapore’s healthcare setting. Overall, this study aims to further understand the perceptions of the respondents, and discuss the underlying concerns that healthcare professionals may have towards the technology, and to identify current complex oral medication regimens that may potentially be simplified by 3D printing.

## 2. Materials and Methods

### 2.1. Design

Our qualitative, descriptive, single-centered survey aimed to understand the perceptions of healthcare professionals regarding 3D printed tablets. The cross-sectional survey was designed based on clinical applications of 3D printing that were suggested in 2015 by Khaled et al. [11] and in 2020 by Awad et al. [12] and Jacob et al. [13]. Potential applications were also considered and discussed within the study team that was made up of pharmacists, innovators, and members of the 3D printing industry. A brief preamble on the introduction and clinical applicability of 3D printed tablets was drafted and included at the start of the survey. The questionnaire was designed to gather respondents’ perceptions of specific clinical applications. Open-ended questions were provided to identify both concerns and possible applications of this technology. This survey was conducted with a web-based questionnaire that targeted healthcare professionals (doctors and pharmacists) at Tan Tock Seng Hospital in Singapore (TTSH).

### 2.2. Setting

The study was conducted at TTSH in Singapore, a 1700-bed university hospital, in collaboration with Craft Health Pte Ltd. Singapore a local pharmaceutical drug delivery company that utilizes 3D printing technologies to simplify drug delivery. The survey was sent to approximately 500 doctors who were involved in the care of patients in the disciplines of general medicine, cardiology, infectious diseases, neurology and geriatric medicine, and to 130 pharmacists at TTSH.

### 2.3. Data Collection and Analysis

The questionnaire was distributed electronically via email to pharmacists and doctors at TTSH between December 2020 to February 2021. Ethics approval was obtained, with an approval ID of DSRB 2020/01275. Ten 5-point Likert scale statements were included, and participants were asked to rate their level of agreement with each statement, ranging from “Strongly Disagree” to “Strongly Agree”. The participants were also asked three open-ended questions and asked to share their suggestions and concerns. The questionnaire was subdivided into three areas: perceptions toward 3D printed tablets, preferences regarding the prescription of 3D printed tablets, and areas of concern.

A simple data analysis, based on qualitative observations, was then performed. The survey questions are set out in Appendix B.

## 3. Results and Discussion

### 3.1. Demographics of Respondents

**Figure 1:**
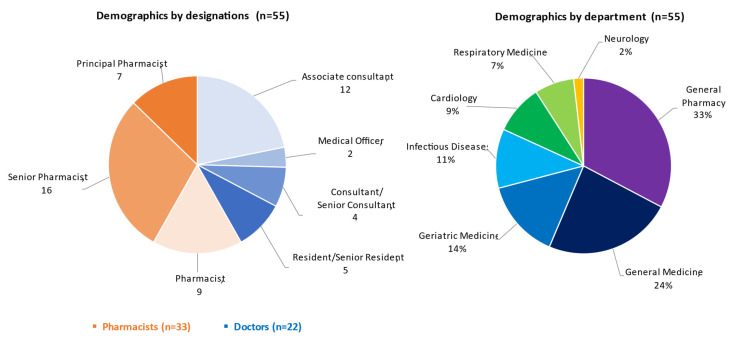
Demographics of survey respondents (*n* = 55).

As shown in Figure 1, a total of 55 healthcare professionals (22 doctors and 33 pharmacists) from seven departments, with various designations and experience levelsresponded.

### 3.2. Survey Results

**Figure 2:**
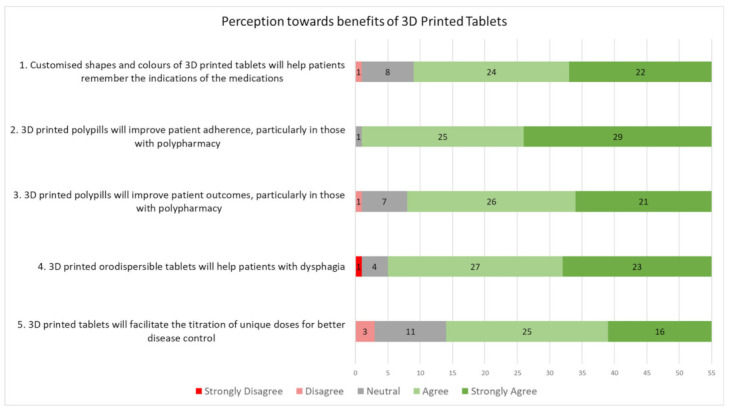
Responses to statements on the benefits of 3D printed tablets.

**Figure 3:**
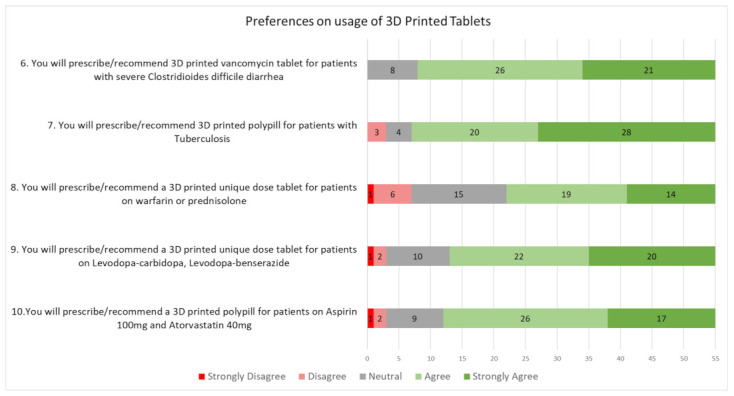
Responses to statements about preferences on usage of 3D printed tablets.

The first five statements of the questionnaire (Figure 2), were about the respondents’ perceptions of the benefits of 3D printed tablets. The next five statements, as shown in Figure 3, were about the respondents’ preferences in prescribing 3D printed tablets. Overall, a majority of healthcare professionals surveyed held a positive perception of 3D printed tablets. As shown in Figure 2, more than 70% (38 out of 55) of the healthcare professionals agreed with the benefit mentioned in each statement. Similarly, as shown in Figure 3, when presented with several possible preferences on usage of 3D printed tablets, a majority of the respondents (>60%) agreed that they would prescribe them, while a few of the respondents stated reservations or disagreed with the statement(s). This indicated a general acceptance by local healthcare professionals of the use of 3D printed tablets in public hospitals in Singapore.

### 3.3. General Perceptions toward 3D Printed Tablets

Most of the respondents agreed with 4, “Customised shapes and colours of 3D printed tablets will help patients remember the indications of the medications”, with 84% of the respondents (46 out of 55) being in agreement, 14% of the respondents (8 out of 55) being neutral, and 2% of the respondents (1 out of 55) disagreeing with the statement. Similarly, 90% of the respondents (50 out of 55)”agreed with, ‘3D printed orodispersible tablets would help patients with dysphagia”, with 7% of the respondents (4 out of 55) being neutral and 2% of the respondents (1 out of 55) strongly disagreeing.

Tt was interesting to note that, “3D printed polypills will improve patient adherence, particularly in those with polypharmacy”, was the most widely agreed upon statement, with 98% of the respondents (54 out of 55) choosing either “agree” or “strongly agree” and the remaining 2% of the respondents (1 out of 55) choosing “neutral”. Patient adherence is a challenge [3], especially for patients who need to take more than five medications a day (also known as polypharmacy) at different frequencies before and after consumptions of food. By combining different drugs into one tablet, 3D printed polypills can help to reduce the number of tablets that a patient needs to take. This key advantage is recognized by healthcare professionals.

However, the respondents were more uncertain whether the polypills would improve patient outcomes, compared with improving adherence. Fifteen percent of the respondents (8 out of 55) did not agree that 3D printed polypills would improve patient outcomes, while only 2% of the respondents (1 out of 55) did not agree that patient adherence would be improved. This could be attributed to different confounding factors that affect patient outcomes. Typically, an increase in medication adherence implies that a patient would receive the intended benefits of the medication, leading to better clinical outcomes [14]. Furthermore, there have been studies showing that improvements in medication adherence occurs, by up to 26%, when there is a reduction in pill burden [15,16,17].

It was also noted thatfor, “3D Printed tablets will facilitate the titration of unique doses for better disease control”, a greater proportion of respondents disagreed or were neutral, compared with the other statements, with 5% of the respondents (3 out of 55) disagreeing and 20% of the respondents (11 out of 55) remaining neutral. Of the three respondents who disagreed, two raised concerns regarding the titration of doses, as per the following quotations: 

Concern 1: *“If medicines need to be titrated or stopped, there would be wastage of 3D printed pills.”*

Concern 2: *“How would titration look like, if the tablet is a combination of multiple drugs?”*

These concerns suggested that the respondents may not have fully comprehended how 3D printing is able to facilitate dose titration. Drug titration refers to adjusting a medication dosage, based on pharmacokinetic and pharmacodynamic factors, to achieve the optimal clinical response from a patient. Drug titration is highly personalized and commonly used for drugs with a narrow therapeutic index [18]. Currently, drug titration requires the expertise of a healthcare provider in adjusting dosages appropriately. With 3D printing technology, changes in dosage can easily be achieved by changing the geometry of a tablet, e.g., its size or shape. Hence, a tablet’s content can be printed precisely with the exact required dosage, without the need to make further adjustments, such as tablet-breaking. In addition, 3D printing allows for the on-demand printing of small batches of tablets. Therefore, unique applications for therapies that require individualized dosages can be better facilitated, reducing drug wastage and error.

Generally, polypills should be prescribed for patients who do not require frequent daily variations in dosages. Unlike fixed-dose combination (FDC) pills (polypills that are currently mass-manufactured by conventional manufacturing processes), polypills produced by 3D printing technologies have the potential to tailor individualised dosages The required dosage for each drug can be determined prior to the combination of the drugs in a single 3D-printed polypill, overcoming the limitation and complexity of titrating an FDC pill.

### 3.4. Preferences on Prescribing 3D Printed Tablets

When shown several indications and asked if they would prescribe 3D printed tablets for their patients, the respondents’ answers varied according to the type of drug or disease indicated. The difference in responses could be due either to different understandings of the various drugs indicated or to differences in preferences about the usage of 3D printing. Table A2 (Appendix A) elaborates on the indications mentioned in the survey and showcases the reasons why 3D printed tablets may be helpful.

It was also observed that there seemed to be greater acceptance of single drugs in 3D printed tablets, compared with combinations of drugs in a single tablet or drugs that require personalized dosages. For instance, it was noted that for statement 9, “You will prescribe/recommend 3D printed vancomycin tablet for patients with severe *Clostridioides difficile* diarrhoea”, no respondents disagreed. However, there were more respondents that disagreed or were neutral for statements 10 to 13, indicating the variation in prescribers’ acceptance of 3D printing technology. This variation could be due to the following concerns, as raised by one of the respondents who disagreed with statement 10:

Concern 3: *“If there is a side effect from the combination tablet, it may be difficult to isolate and identify the causative agent (for example: the aforementioned suggestion to combine 4 TB [tuberculosis] medications into a single pill).”*

Finally, when asked about what other constituents of 3D printed polypills might potentially be printed, the respondents mentioned oral hypoglycemic agents, ant-hypertensives, anti-platelets, beta-blockers, and supplements, including calcium and iron. The respondents indicated that medical conditions that may benefit from 3D printed tablets included diabetes, chronic kidney disease, myocardial infarction, heart failure, hypertension, stroke, H. pylori infection, Parkinson’s disease, and dementia.

### 3.5. Areas of Concern

Only 9% of the respondents (5 out of 55) failed to raise a concern. Overall, the concerns can be grouped into three broad categories: formulation issues, administrative concerns, and manufacturing and regulatory concerns.

#### 3.5.1. Formulation Issues

Some of the main concerns raised by respondents included the stability and bioequivalence of the tablets, the size of the tablets, drug interactions, and compatibility issues (Table 1).

Twenty-two percent of the respondents (12 out of 55) mentioned stability and/or bioequivalence as a concern. The safety and efficacy of tablets are of utmost importance. Accordingly, as with conventional tablets, 3D printed tablets must be subjected to stability tests before commercialization. The pharmacokinetics and release profile of the drugs must also be tested to evaluate the drugs for bioequivalence to medicines that are already prescribed in clinics. Such testing will ensure that 3D printed tablets are safe to consume. The packaging of 3D printed tablets also plays an important role and should be suitable for protecting the tablets against harsh conditions.

Seven percent of the respondents (4 out of 55) mentioned the size of the tablets as a concern. They were concerned that polypills with multiple drugs and components would result in a bigger tablet, which may hinder patients from ingesting it. Generally, the size of a tablet is dependent on the potency of the active pharmaceutical ingredient (API), as well as the excipient-to-active-ingredient ratio. However, compared with conventional tablets, which often require bulking agents to aid in tableting, 3D printed tablets require lesser excipients and can achieve a higher loading dose without significant increase in tablet size. Accordingly, the size of the 3D-printed tablets would not be significantly affected even with multiple types of dosages or ingredients. In addition, for patients with dysphagia, there is the possibility of printing orodispersible polypills [19].

Twenty percent of the respondents (11 out of 55) raised concerns regarding drug interaction, with a focus on the compatibility and potential adverse effects of the drugs used. Generally, in the case of drugs that are not contraindicated, drug interactions can be managed using 3D printing and tablet design. For instance, blank layers (containing no active ingredients) can be used to physically separate drugs from one another. The time of release of each individual drug can also be tuneable, releasing one drug before another in a form of temporal separation, to reduce the interactions between multiple drugs. Hence, with flexibility in adjusting the designs and formulations, 3D printing can prevent harmful drug interactions. Other than the printing parameters, the excipients and active ingredients used in a formulation should conform to prevailing good manufacturing practice (GMP) with the excipients from the FDA’s “Generally Regarded as Safe” (GRAS) list, or adapted to the target market’s regulatory requirements.

#### 3.5.2. Administrative Concerns

Respondents’ concerns about administrative aspects related to 3D printed tablets mainly fell into the following two sub-categories: medication reconciliation and the reduced health literacy of patients (Table 2). Eighteen percent of the respondents (10 out of 55) expressed concerns regarding information flow and proper documentation for 3D printed tablets. They were concerned that 3D printed tablets might be confusing for patients and healthcare professionals in different health institutions.

As emphasized by a few of the respondents, good documentation and a standardized workflow are needed to ensure the proper flow of information to various healthcare providers, to allow for easy medication reconciliation and proper product identification. One way to do so would be to print a unique barcode or QR code on the packaging of the printed tablets. The barcode would contain drug information and the production date, as well as prescription instructions. When dispensed, the barcode would be scanned and this information would be entered into the patient’s healthcare database, which could be accessed by various healthcare providers. Patients would also be able to access this information through the same barcode or QR code, which would enable them to distinguish between different batches or types of pills, if needed.

In addition to the proposed workflow mentioned previously, adequate counseling by pharmacists remains an important aspect of the drug dispensing process in order to enable patients to have improved health literacy. As 3D printed tablets become more common and accepted by the public, patients will have increased awareness of the types of medicines they are taking.

#### 3.5.3. Manufacturing and Regulatory Concerns

Many concerns were raised about the manufacturing and regulatory aspects of 3D printed tablets. The concerns generally fell into the following subcategories: quality control and assurance, time, and costs, as well as regulatory challenges (Table 3).

Eighteen percent of the respondents (10 out of 55) raised quality control and quality assurance (QA/QC) as concerns. QA/QC are important in the manufacture of 3D printed tablets. In order to assure end-users that the 3D printed tablets are of high and consistent quality, 3D printing companies should conform to recognized standardized formats of manufacturing, such as those of good manufacturing practice (GMP) and the International Organization for Standardization (ISO). This standardization would mean that a company has proper quality management systems in place for data recording and audit purposes. In addition, other measures to improve consistency between batches of 3D printed tablets would include sourcing GMP-grade excipients and active ingredients from companies that are GMP-certified. The consistency of the active ingredient is dependent on its manufacturing process and the logistics chain; hence, sourcing from GMP-certified companies would give added assurance that the raw ingredients are of good quality.

Cross-contamination is another serious issue. As mentioned, the production facility for both commercial and in-house printing should be a clean room that is GMP/ISO-certified, with validated cleaning protocols in place to reduce contamination. Appropriate measures should be put into place, such as only printing one type of formulation at any one time.

Other key concerns are the manufacturing costs and the time needed for 3D printed tablets. Twenty-nine percent of the respondents (16 out of 55) raised concerns regarding costs, and 15% of the respondents (8 out of 55) raised concerns regarding turnover times. Generally, they were concerned about the tablets’ affordability for patients and the waiting times for patients to get their medications.

The cost of a medication depends on many factors, such as the population distribution and the active ingredients used. From a manufacturing standpoint, 3D printing allows costs to be reduced, especially for orphan diseases or treatments that require unique dosages, because of the ability of 3D printing to print on-demand in small batches. In addition, 3D printing is potentially more cost effective for the mass customization of tablets, compared with conventional manufacturing processes. Generally, conventional manufacturing processes are suited for mass-producing only one product at a time. On the other hand, 3D printing is more versatile and allows for the production of multiple products (with variations in dosages or shapes, for example) within the same production batch. In addition, 3D printing has a comparably shorter manufacturing line, facilitating quick transition between different formulations and adaptation to the ever-changing healthcare landscape.

The costs of 3D printers vary widely, depending on the type of 3D printing technique that is used. In general, among the printers with 3D printing techniques for pharmaceuticals, BJ printers cost the most, followed by FDM printers and SSE printers. The cost of these printers ranges widely, from USD 500 to USD 150,000. However, this initial capital investment in a 3D printer would likely be nowhere close to the cost of a conventional rotary tablet press, which could easily be almost ten times the cost of a 3D printer. In addition, the floor space required by a 3D printer is much less than that required for a conventional rotary tablet press. The compact size of a 3D printer makes it suitable for use in a point-of-care setting, where pharmaceuticals are produced at the hospital. Beyond the initial capital investment, the recurring costs of a 3D printed tablet would be comparable to those of a conventional rotary tablet press, as the bulk of the recurring costs would be the costs of raw ingredients. Based on available research, the raw ingredients used for 3D printed tablets are the common excipients that are already used for conventional rotary tablet presses. The quality control solutions for a conventional tablet are the same as those of a 3D printed tablet; therefore, quality control will not result in additional costs in implementing 3D printing in pharmaceuticals. Finally, the real-time on-demand approach with 3D printed pharmaceuticals reduces wastage due to expiry or changes in required doses. Based on the annual report of a local pharmaceutical distribution company in Singapore, the cost of goods written off in 2021 consumed approximately 6.9% of the company’s profit [20]. It is difficult to provide an accurate estimate of the setting-up costs of a pharmaceutical 3D printing facility, as these costs may vary widely in different settings or countries, and also depend on the directions of business discussions. Hence, further studies need to be conducted to determine the actual tangible cost effectiveness of a point-of-care 3D printed tablet and the required production facility.

Improvements in patient outcomes, with the increase in medication adherence due to taking polypills, may also potentially lead to cost savings for patients, as there will be reduced risks of hospitalization and/or a reduced need to utilize healthcare services [21].

Turnaround time should not be a concern. Generally, for mass-manufactured tablets such as polypills, the production time is relatively comparable to conventional manufacturing methods. In an interview with Craft Health’s co-founder, it was noted that Craft Health has a throughput of 3000 to 5000 tablets per day from a single 3D printer [22].

For point-of-care, print-on-demand pills, such as those with personalized dosages, the turnaround time can be relatively fast, especially if such pills are printed in-house in hospitals. This can be achieved with the production of GMP-certified printers and specialized training for pharmacists. With the development of the first GMP-certified printer by FabRx in 2020 [23], and with other companies working to produce GMP-certified printers, this technology will become more accessible for hospitals in the near future.

Currently, as the 3D printing of pharmaceuticals is still considered to be a new industry, the regulatory process remains a challenge in Singapore. There are no guidelines by the Health Science Authority (HSA) for the approval of 3D printed tablets. However, the United States Food and Drug Administration’s Center of Drug Evaluation and Research (CDER) is currently developing regulatory guidelines for the 3D printing of pharmaceuticals [24]. With the approval of Spritam^®^ and the continuous advancements in 3D printing technologies, 3D printing of pharmaceuticals is expected to gain more traction in the next few years, facilitating the development of regulatory guidelines in Singapore and other regions. Moreover, 3D printing may bring advantages in improving the efficiency of drug development and lowering the costs of trials, as it can produce smaller batches of drugs in short timeframes [25]. There remains a need for regulatory bodies, 3D printing companies, and hospitals to work closely together to facilitate the implementation of this technology which has the great potential to accelerate new drug development and address current clinical challenges.

### 3.6. Limitations of Study

Our limited sample size may not have reflected the true perceptions and concerns of the respondent healthcare professionals. As 3D printed tablets are relatively new, some survey respondents may not have a good understanding of the technology, which could have affected their responses. Future studies could be based on formulation issues, administrative issues, manufacturing processes, cost effectiveness, and the regulatory concerns that can further drive the clinical applicability of 3D printing technology.

## 4. Conclusions

Overall, the respondent healthcare professionals indicated a positive perception of 3D printed tablets. This result is essential and promising for the future adoption of 3D printed tablets in local hospitals. However, as 3D printing technology is still emerging, it is important to understand the perspectives and concerns of the prescribers, regulators and the technology providers to advance the benefits of this technology from bench to bedside.

## Figures and Tables

**Table 1:** Concerns raised by healthcare professionals regarding the 3D printed tablets.

Subcategory	Respondents’ Comments. (Comments Have Been Edited Slightly for Grammatical Clarity.)
Stability and bioequivalence	*“Stability of tablets—How is the expiry date determined? What if the patient does not keep the tablet in the recommended conditions?”*
	*“How is bioequivalence ensured?”*
	*“How would changes in release profile affect the pharmacokinetics & pharmacodynamics of the drug”*
Size of tablets	*“What would the size of the tablet be like if the patient requires many medications?”*
	*“Would the size of tablets be suitable and crushable for patients with dysphagia?”*
Drug interactions	*“Would there be drug interaction issues in the polypills created?”*
	*“It may be hard to know which component is causing side effects or allergic reactions, especially in polypills.”*

**Table 2:** Concerns raised by healthcare professionals in the administrative category.

Subcategory	Respondents’ Comments. (Comments Have Been Edited Slightly for Grammatical Clarity.)
Medication reconciliation	*“How would healthcare providers identify the drug content or communicate across healthcare institutions to confirm the drugs the patients are on if each 3D pill is unique to the patient’s requirement?”*
	*“Patients need to be able to distinguish between different polypills collected at different time points. For example: when there are dosage changes but the polypills collected have the same appearance”*
Reduced health literacy of patients	*“There may be reduced patient awareness of the medications that patients are on. There needs to be good documentation of what each customised tab contains.”*
	*“3D printed polypills may potentially reduce patient’s health literacy and incentive to participate in their medical care due to the reliance on prepacked medicine”*

**Table 3:** Concerns raised by healthcare professionals regarding manufacturing processes.

Subcategory	Respondents’ Comments. (Comments have Been Edited Slightly for Grammatical Clarity.)
Quality control and assurance	*“There should be batch standardisation and confidence that for Narrow Therapeutic Index drugs, each product is within reasonable margin of error for product variance”*
	*“Quality assurance of in-house 3D Printing should be ascertained”*
	*“How can cross contamination in the production process be prevented and consistency of dosages and active ingredients be ensured?”*
Manufacturing time and cost for patients	*“Would the cost and time required to obtain the 3D printed pill be practical for patients requiring frequent titration of medication?”*
	*“How fast is the turnaround time, to prevent interruption of therapy?”*
	*“Would it be time consuming to 3D print large quantities of medicines?”*
Regulatory challenges	*“A long lead time and extensive testing for regulatory submission is needed. Would a change in formulation require application with HSA under the New Drug Application route?”*

**Figure A1:**
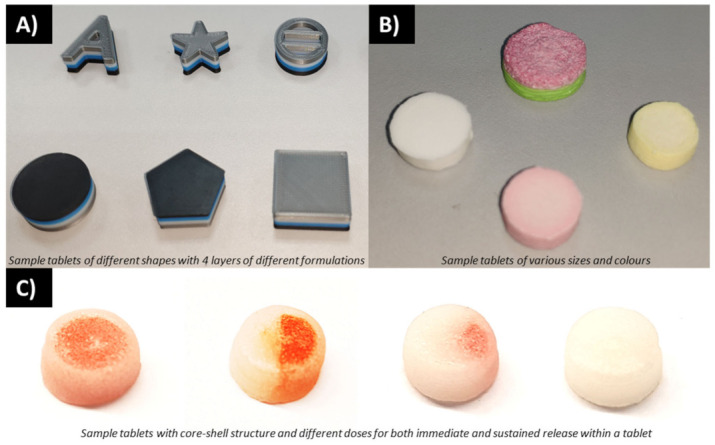
Image of various 3D printed tablets. (Credit: Craft Health Pte. Ltd., Singapore).

## Data Availability

The data presented in this study are available on request from the corresponding author.

## Appendix A

**Table A1:** Impact of 3D printing and current advances in technology.

Unique Features of 3D Printed Tablets	Impact/Advantages	Examples of Applications	3DP Technology Used
Customised colors and shapes	Helps patients to identify and remember indications of medications [26] Improves medicine acceptability [7,8]	Tablets with braille and moon patterns for visually impaired patients [12]	Selective laser sintering (SLS)
		Chewable tablets [27]/gummies [28] shaped like candy for pediatric use	Fused deposition Modeling [27] Embedded 3D printing [28]
Polypills	Combines multiple drugs, with different release profiles into 1 pill. Reduces patient’s pill burden Improves patient’s adherence and outcomes	Four-in-one polypill with multiple release profiles [29]	Semi-solid extrusion
		Polypills with bespoke release patterns for multiple drugs [30]	Fused deposition Modeling
		Multi-layered polypill containing six drugs [31]	Stereolithographic (SLA)
Oro-dispersible tablets	Ensures accurate dosing compared to liquid solutions Helps patients with dysphagia, including children and the elderly	Spritam^®^, first commercialised 3D printed drug [32]	Binder jet 3D Printing
		Oro-dispersible pills in cartoon shapes for pediatric applications [33]	Color jet 3D Printing
Customized doses	Suitable for patients who require constant changes in medication dose Facilitates titration of unique doses; better disease control	First clinical trial using chewable isoleucine tablets with varying personalised dosages and flavors [34]	Semi-solid extrusion

**Table A2:** Current state of treatment for diseases/drugs mentioned in questionnaire and the improvements that 3D printing can bring.

Indication	Drugs	Current State of Treatment	Significance of 3D Printed Tablets
*Clostridioides difficile* Diarrhea	Vancomycin (antibiotic)	Ingestion of the liquid form of vancomycin which is unpleasant tasting and prone to inaccurate dosages. Capsule forms of vancomycin are available, but the low demand and resulting high costs pose an economical barrier for patients [35].	3D printing can change the dosage forms of conventional liquid medications to solid oral forms, ensuring accurate dosing and improving patients’ adherence. 3D printing is suited for small batch manufacturing, keeping costs low for “low-demand” or orphan drugs.
Tuberculosis	Combination of drugs must be taken. These include: Rifampicin, Isoniazid, Pyrazinamide, Ethambutol, and Pyridoxine [36,37]	Patients are required to follow a strict regimen of taking up to 11 pills [38] daily for a few months In Singapore, patients are under directly observed therapy (DOT) where they are required to take these pills supervised.	3D printing can produce polypills that combine various drugs into one tablet. This would greatly reduce the number of pills patients need to take daily and reduce the associated monitoring time by clinicians and caregivers.
Anticoagulation	Warfarin (anticoagulant)	Daily doses are dependent on the international normalised ratio (INR) in patients’ blood and can range from 1 mg to 10 mg, varying from person to person [39,40]. Current tablets have fixed dosing with different strengths. Hence, patients need to break the tablets and “mix-and-match” to achieve the daily required dosage.	3D printing can print on-demand with the accurate dosing amount required in the pill. Patients would not need to “mix-and-match” from fixed dose tablets, thereby reducing the chances of inaccurate dosing and medication error.
Anti-inflammation	Prednisolone (corticosteroid)	Dosage required differs from person to person. Patients are often given a strong dose at the start of treatment. As treatment progresses, the dosage is adjusted and gradually reduced [41].	
Parkinson’s disease	Levodopa-carbidopa/Levodopa-benserazide	Medications to manage symptoms are only effective for short durations [42]. Patients must take tablets up to five times a day. Wide range of dosages, which also differ for person to person	3D printing can print sustained-release tablets and personalize dosages for patients. This would reduce the frequency of pill-taking.
Cardiovascular	Aspirin and Atorvastatin	Common combination for many cardiovascular diseases [43].	Drugs can be combined into a generic polypill for easy prescription; reducing the overall number of drugs.

## Appendix B. Survey Questions

Preferences of Healthcare Professionals in 3D printed tablets: A pilot study

1. What is 3D Printing?

3-Dimensional (3D) printing, also known as additive manufacturing (AM), fabricates a structure via deposition, binding or polymerisation of materials in successive layers until the complete object is created. It was incepted in the mid-1980′s and has gained increasing amount of traction for its applications over the last 15 years. The rapid prototyping nature of 3D printing allows small batch manufacturing of different objects to be done quickly, without the use of a mould as per traditional manufacturing.

2. Has 3D Printing been used in pharmaceuticals?

The efforts and advances of 3D printing in the field of pharmaceutics have recently culminated in the US Food and Drug Administration (FDA) approval of the world’s first 3D printed orodispersible tablet SPRITAM^®^ (3D printed levetiracetam) in 2015.

3. What are the advantages of 3D printing in pharmaceuticals?

3D printing allows the improvement of current pharmaceutical dosage forms through complex and customised dosage forms which are not cost-effective or otherwise impossible with traditional manufacturing. These advantages include:

- Combination of various medicines or supplements into a single polypill
- Reformulation of various medicines into a new controlled release profile (immediate/sustained/enteric/delayed/ orodispersible controlled release forms)
- Rapid small batch manufacturing of new formulations
- 3D printing of new shapes, geometries or colours for easier recognition

4. Patient centric pharmaceutical dosage forms

With the ability to produce complex and customised dosage forms, 3D printed tablets provide opportunities to design patient centric pharmaceutical dosage forms. Customized shapes and colours help patients to remember their indication as well as when to take the medicine. Some specific examples include:

(1) Medicines which are unavailable in a suitable tablet form can be 3D printed quickly. Oro-dispersible tablets can also be created for patients with dysphagia.
(2) Patients on unique doses who require breaking the usual mass-produced dosage forms (e.g., breaking a 2mg table to achieve a daily dose of 3.5 mg) can now obtain a 3D printed tablet with a personalized dose.
(3) Embossing designs have the potential to include Braille and can help visually impaired patients to identify the correct medications to take.
(4) Polypills can be designed where up to 5 different active pharmaceutical ingredients with immediate release and sustained release profiles all in one.

This survey aims to identify the disease states, patient population and the medicines that doctors and pharmacists will find application for 3D printing of tablets. In addition, we aim to ascertain the perceived benefits and concerns about this technology.

*Please respond to the questions using the 5-point Likert skill unless otherwise specified.*

1. What is your profession?				
(a) Doctor	(b) Pharmacist			

2. What is your current designation?					
(a) Associate consultant and above	(b) Resident/Senior resident	(c) Medical Officer	(d) Principal pharmacist	(e) Senior pharmacist	(f) Pharmacist

3. What is your specialty?				
(a) General Medicine	(b) Cardiology	(c) Infectious diseases	(d) Neurology	(e) Geriatric Medicine
(f) Respiratory medicine	(g) Pharmacy			

4. Customized shapes and colours of 3D printed tablets will help patients remember the indications of the medicines. *For instance, different colours and patterns may be used in the identification and differentiation of tablets that look alike. Different shapes may also be 3D printed to appeal to certain demographics such as paediatric patients.*				
(a) Strongly disagree	(b) Disagree	(c) Neutral	(d) Agree	(e) Strongly agree

5. 3D printed polypills will improve patient adherence particularly in those with polypharmacy. *For instance, multiple medicines may be combined into a single tablet. In such cases, the patient may be able to reduce the number of pills to be taken daily.*				
(a) Strongly disagree	(b) Disagree	(c) Neutral	(d) Agree	(e) Strongly agree

6. 3D printed polypills will improve patient outcomes particularly in those with polypharmacy. *This may be associated with the reduction in the number of pills to be taken daily, allowing easier persuasion of the patient to take their medicines and to reduce episodes of forgetfulness.*				
(a) Strongly disagree	(b) Disagree	(c) Neutral	(d) Agree	(e) Strongly agree

7. 3D printed orodispersible tablets will help patients with dysphagia (difficulty in swallowing). *Orodispersible tablets can dissolve quickly with limited water, or in the patient’s mouth, reducing the need to swallow. Tablets may be 3D printed as an orodispersible formulation depending on the medication required.*				
(a) Strongly disagree	(b) Disagree	(c) Neutral	(d) Agree	(e) Strongly agree

8. 3D printed tablets will facilitate the titration of unique doses for better disease control. *For instance, half doses of medicines may be 3D printed instead of the current practice of splitting the tablet or taking the tablet on alternate days. Also, the dosing of medicine may be personalized to the individual depending on the requirements for the condition, under the prescribing doctor’s supervision.*				
(a) Strongly disagree	(b) Disagree	(c) Neutral	(d) Agree	(e) Strongly agree

9. You will prescribe/recommend 3D printed vancomycin tablet for patients with severe *Clostridiodes difficile* diarrhea. *Oral vancomycin tablet/capsule is currently not available in Singapore. The required dose of vancomycin may be 3D printed into an oral tablet for the individual patient.*				
(a) Strongly disagree	(b) Disagree	(c) Neutral	(d) Agree	(e) Strongly agree

10. You will prescribe/recommend a 3D printed polypill for patients with Tuberculosis. *The typical regimen for Tuberculosis treatment involves 4 different medications to be taken daily, and medication adherence is important in controlling the condition and preventing the risk of spreading and development of resistance. 3D printing allows all the medications to be formulated in a single tablet.*				
(a) Strongly disagree	(b) Disagree	(c) Neutral	(d) Agree	(e) Strongly agree

11. You will prescribe/recommend a 3D printed unique dose tablet for patients on warfarin or prednisolone. *Warfarin and prednisolone dosing typically require dose titration depending on INR testing or the disease condition. 3D printed tablets allow for the exact dose required to be produced for the individual patient.*				
(a) Strongly disagree	(b) Disagree	(c) Neutral	(d) Agree	(e) Strongly agree

12. You will prescribe/recommend a 3D printed unique dose tablet for patients on Levodopa-carbidopa, Levodopa-benserazide. *The current dose and dosing frequencies of these medicines may be complex (e.g., requires taking half tablets or up to five times a day.) This medicine schedule could be personalized using 3D printing. This may be done through the control of different drug release within the same tablet, such as immediate, sustained or delayed release formulations within one tablet.*				
(f) Strongly disagree	(g) Disagree	(h) Neutral	(i) Agree	(j) Strongly agree

13. You will prescribe/recommend a 3D printed polypill for patients on Aspirin 100mg and Atorvastatin 40mg. *These medications may be 3D printed into a single, fixed dose combination tablet.*				
(k) Strongly disagree	(l) Disagree	(m) Neutral	(n) Agree	(o) Strongly agree

14. List any concerns that you have on 3D printing of tablets

15. List other medical conditions that you think 3D printing tablets can be helpful

16. List any concerns that you have on 3D printing of tablets
